# Application of loop mediated isothermal amplification (LAMP) assays for the detection of *Onchocerca volvulus*, *Loa loa* and *Mansonella perstans* in humans and vectors

**DOI:** 10.3389/fitd.2022.1016176

**Published:** 2023-01-09

**Authors:** Glory Ngongeh Amambo, Ngong Innocentia, Raphael Awah Abong, Fanny Fri Fombad, Abdel Jelil Njouendou, Franck Nietcho, Relindis Ekanya, Chi Anizette Kien, Rene Ebai, Benjamin Lenz, Manuel Ritter, Mathias Eyong Esum, Kebede Deribe, Jerome Fru Cho, Amuam Andrew Beng, Peter Ivo Enyong, Zhiru Li, Marc P. Hübner, Kenneth Pfarr, Achim Hoerauf, Clotilde Carlow, Samuel Wanji

**Affiliations:** 1 Parasites and Vector Research Unit (PAVRU), Department of Microbiology and Parasitology, University of Buea, Buea, Cameroon; 2 Research Foundation in Tropical Diseases and Environment (REFOTDE), Buea, Cameroon; 3 Department of Biomedical Science, Faculty of Health Sciences, University of Buea, Buea, Cameroon; 4 Institute of Medical Microbiology, Immunology and Parasitology, University Hospital Bonn, Bonn, Germany; 5 Global Health and Infection Department, Brighton and Sussex Medical School, Brighton, United Kingdom; 6 School of Public Health, Addis Ababa University, Addis Ababa, Ethiopia,; 7 New England Biolabs, Ipswich, MA, United States; 8 German Center for Infection Research (DZIF), Partner Site Bonn-Cologne, Bonn, Germany; 9 German-West African Centre for Global Health and Pandemic Prevention (G-WAC), Partner Site Bonn, Bonn, Germany

**Keywords:** *Onchocerca volvulus*, *Loa loa*, *Mansonella perstans*, LAMP (loop mediated isothermal amplification), diagnostic test

## Abstract

Conventional diagnosis of filarial infections is based on morphological identification of microfilariae using light microscopy and requires considerable expertise, is time-consuming, and can be subjective. Loop-mediated isothermal amplification (LAMP) has advantages over microscopy or PCR because of its operational simplicity, rapidity and versatility of readout options. LAMP assays represent a major step forward in improved filarial diagnostic tools suitable for low resource settings and field applicability. The study goal was to retrospectively evaluate the performance and suitability of the O-150, RF4, and Mp419 LAMP assays for diagnosing *Onchocerca volvulus*, *Loa loa* and *Mansonella perstans* infections, respectively, in humans and vectors under experimental and natural field conditions. Surveys were conducted in four health districts of Cameroon using skin snip and thick blood film methods to detect skin (*O. volvulus*) and blood (*L. loa* and *M. perstans*) dwelling microfilaria in humans. Engorged vectors (*Simulium spp*., *Chrysops spp*., and *Culicoides* spp.) were evaluated by LAMP. Dissected, wild-caught vectors were also analyzed. LAMP showed a prevalence of 40.4% (*O. volvulus*), 17.8% (*L. loa*) and 36.6% (*M. perstans*) versus 20.6% (*O. volvulus*), 17.4% (*L. loa*) and 33.8% (*M. perstans*) with microscopy. *Simulium* spp. were dissected for microscopy and pooled for LAMP. The O-150 LAMP assay infection rate was 4.3% versus 4.1% by microscopy. *Chrysops* spp. were dissected and analyzed individually in the LAMP assay. The RF4 LAMP assay infection rate was 23.5% versus 3.3% with microscopy. The RF4 LAMP assay also detected parasites in *Chrysops* spp. fed on low microfilaremic volunteers. The Mp419 LAMP assay infection rate was 0.2% for *C. milnei* and 0.04% for *C. grahamii*, while three other species were LAMP-negative. The sensitivity, species specificity, rapidity and ease of its use of these filarial LAMP assays, and validation of their performance in the field support use as alternatives to microscopy as diagnostic and surveillance tools in global health programs aimed to eliminate onchocerciasis.

## 1 Introduction

Onchocerciasis, a.k.a. river blindness, is a debilitating vector-borne (*Simulium* spp.) disease caused by *Onchocerca. volvulus* (1, 2). The disease can present as dermatitis, skin nodules and ocular lesions, resulting from the migration of the first stage larvae (microfilariae, mf) through the skin and eyes, leading to an intense, papular dermatitis associated, itch and/or even severe ocular damage that can result in blindness (2). The disease is endemic in Africa, Yemen and Latin America; affecting over 37 million people of which 99% live in Africa (1). The mass drug administration (MDA) programs to control onchocerciasis have implemented community-wide treatment of the endemic populations with ivermectin (CDTI). However, despite more than 20 years of CDTI, onchocerciasis is still a major public health and socioeconomic problem for several endemic countries, including Cameroon (1).

In many areas, onchocerciasis MDA programs have run for several years and are entering a period where important decisions as to their continuation need to be made. In order to evaluate their success, certify elimination and guide the decision to stop MDA, careful monitoring of infection in human hosts and vectors with sensitive and specific diagnostic tools is required. Conventionally, diagnosis is based on identification of microfilariae (mf, first stage larvae) in skin biopsies (onchocerciasis) or blood (for the related co-endemic infections mansonelliosis and loiasis) as well as insect vectors using light microscopy (1). To achieve the World Health Organization goal of interrupting transmission of onchocerciasis in support of the UN Sustainable Development Goals (1), MDA should be extended to all endemic regions, including those that have been exempted due to co-endemicity with loiasis. The latter requires new diagnostics that can rapidly identify and differentiate co-endemic filarial nematode infections, e.g., *Loa loa* or *Mansonella perstans*.

The filarial worm *L. Loa* (African eye worm) causes loiasis and can be co-endemic with onchocerciasis in Cameroon, Central African Republic, Democratic Republic of Congo, Gabon, Nigeria, and the Republic of Congo, (3). The parasite is transmitted to humans by the tabanid flies *Chrysops silacea* and *C. dimidiate* (4, 5) in rainforest and savannah areas of Central and Western Africa (3, 6); where circa 3-13 million people are affected (7). Despite transient, localized angioedema and sub-conjunctival migration of the adult worm (8), *L. loa* infection has been considered a benign condition. However, ivermectin treatment of persons infected/co-infected with *L. loa*, especially those with high microfilarial loads (>30,000 mf per milliliter, mf/mL), may experience severe adverse events (encephalopathy) (9–11). Hence, MDA cannot be safely carried out in these areas of co-endemicity (12–14).

Mansonellosis is a filarial infection caused by *M. perstans*, *Mansonella*. sp. DEUX, *M. ozzardi*, *M. streptocerca* and *M. rodhaini*. *M. perstans* has the widest geographic distribution; 33 countries in sub-Saharan Africa and in South America (15–17). Mansonellosis is probably the most prevalent of all the human filarial infections with prevalence rates of 3–96% (15, 17, 18), nevertheless, there are no MDA programs for this infection. An estimated 114 million people in Africa are infected with *M. perstans* and 580 million are at risk. Infected individuals may suffer non-specific symptoms of infection: chronic pruritis, fever, headache, joint pain, lymphadenopathy, and rashes (15), while some may also experience ocular lesions due to adult *M. perstans* in the eyes (19). Mansonellosis, particularly due to *M. perstans*, may down regulate immune responses (20), enhancing susceptibility to other infections such as tuberculosis and malaria (21), as well as negatively affecting the efficacy of vaccines (17).

*Mansonella* spp. infection lack specific immunodiagnostic tests (17), but there are several immunological methods for detection of either antibody or antigen for *L. loa* (22) and *O. volvulus* (2). However, low levels of sensitivity (23) and cross-reactivity (24–26) have been reported. Monitoring infections in the vectors is essential for assessing transmission and recrudescence (27). Vector transmission assessments requires microscopic identification of parasites in captured vectors; requiring expertise to morphologically identify the parasites and is a slow process that is not practical for large-scale surveys. Nucleic acid-based assays provide alternative assays with higher sensitivity than parasitological or immunological methods that can be used on samples from humans and vectors.

Polymerase chain reaction (PCR)-based methods have been developed to detect *Mansonella* spp. (28–30), but do not distinguish *M. perstans* from *M. ozzardi* without an additional step of sequencing the amplicon (29), *L. loa* (29, 31), and *O. volvulus* (2, 32, 33). Generally, PCR assays are more sensitive than the conventional parasitological technique, yet they require trained personnel and relatively expensive equipment; which can limit their widespread use (34). A simpler method that circumvents some of the current limitations to specifically diagnose filarial infections in humans and vectors would overcome the technical barriers of in field or low-resource settings,

Loop-mediated isothermal amplification (LAMP) is a simple to perform alternative to PCR that does not require expensive equipment and offers a colorimetric readout particularly suitable for low resource settings (35–37). In addition, LAMP is not as sensitive to inhibitors commonly found in clinical specimens and insects that inhibit the Taq polymerases used in PCR (38). Our consortium has developed highly specific and sensitive LAMP assays for *L. loa* (36), *O. volvulus* (39), and *M. perstans* (40). The present study evaluated the performance and suitability of the *O. volvulus* O-150, *L. loa* RF4 and *M. perstans* Mp419 LAMP assays for the detection of these filarial infections in humans and different vector species under experimental and field conditions.

## 2 Results

### 2.1 Detection of filarial infection in humans using microscopy and LAMP assays

For *O. volvulus* infections, a total of 670 skin snips samples were collected from four health districts (HD) in the Littoral region of Cameroon that were subjected to microscopy and the O-150 LAMP (Table 1). Overall, 138 samples (20.6%) were positive by microscopy, while 271 samples (40.4%) were positive by LAMP. The proportion of microfilaridermia positivity detected by the LAMP assay was significantly higher than by microscopy in all HDs.

For *L. loa* infections, 472 blood samples were collected from 4 HDs of the Littoral region of Cameroon and analyzed by the RF4 LAMP assay (Table 1). Overall, 82 samples (17.4%) were positive by microscopy, while 84 samples (17.8%) were positive by LAMP. The number of samples scored positive by LAMP assay were similar to those scored by microscopy.

For *M. perstans* infections, 725 human blood samples from participants in 4 HDs of the Littoral regions of Cameroon were examined for *M. perstans* infection using microscopy and the Mp419 LAMP assay (Table 1). Overall, 245 samples (33.8%) were positive by microscopy, while 265 samples (36.5%) were positive by LAMP. The number of samples scored positive by LAMP assay were similar to those scored by microscopy.

### 2.2 Overview of the performance characteristics of the O-150, RF4 and Mp419 LAMP assays

The accuracy (sensitivity, specificity, positive predictive value (PPV), negative predictive value (NPV) and the Kappa index) of all three LAMP assays using microscopy as reference diagnostic method were calculated (Table 2). The O-150 LAMP assay detected 133 more *O. volvulus* positive skin snips than microscopy. The RF4 LAMP detected 80/82 microscopy positive samples; 4 microscopy negative samples were RF4 LAMP-positive and 2 microscopy positive samples were negative. The Mp419 LAMP assay detected 28 more positive samples than microscopy; 7 microscopy-positive samples were negative by the LAMP assay.

All three LAMP assays had higher sensitivity and specificity than microscopy. The RF4 and Mp419 LAMP assays had high PPV, while O-150 was intermediate; however, these findings need to be discussed against the fact that mf microscopy in onchocerciasis has a low sensitivity, in particular in hypoendemic settings (see discussion), and a latent class analysis would be helpful to determine the “true” (=higher) prevalence values (41). The NPV for all three LAMP assays was high. The Kappa indices of the RF4 and Mp419 LAMP assays indicated that the LAMP assays are equivalent to microscopy in these endemic settings. As the O-150 LAMP assay was more sensitive than microscopy, the Kappa index was, as expected, lower.

### 2.3 Detection of *O. volvulus* infection in experimentally infected *Simulium* flies using O-150 LAMP

A total of 16 pools (2 flies/pool) of flies that had fed on a volunteer (mf load 50 mf/mL) were analyzed using the colorimetric O-150 LAMP assay. Positive (*O. volvulus* DNA) and negative (or molecular biology grade H_2_O) control samples were included. Of the 16 pools, which consisted of 32 engorged flies, all 16 (100%) were positive by the LAMP assay.

### 2.4 Detection of *L. loa* infection in experimentally infected *Chrysops* flies using LAMP technology

Equal numbers (18) of *Chrysops* flies that had fed on volunteers with low (<10 mf/mL) or high (>30,000 mf/mL) mf load were evaluated with the colorimetric RF4 LAMP assay. Positive (*L. loa* DNA) and negative (molecular biology grade H_2_O) control samples were included. Of the 18 fliesthathadfedonthevolunteerwithlow parasitemia, 88.9% were positive, while 94.4% of the 18 flies that had fed on the volunteer with high parasitemia were positive (Table 3). Detection of infection in flies that fed on the volunteer with low parasitemia was limited to the abdomen up to 7 days post infection, while from day 10 post infection, parasites were also detected in the head and thorax. However, in flies fed on a volunteer with high parasitemia, the LAMP assay detected infection at all-time points in all parts of the flies.

### 2.5 Detection of *M. perstans* infection in experimentally infected *Culicoides* spp. using LAMP technology

*Culicoides* spp. which were fed on two volunteers with microfilarial loads of 80,000 mf/mL and 3,000 mf/mL, respectively, were evaluated using the colorimetric Mp419 LAMP assay. Of the 10 pools comprised of flies which had fed on a volunteer with 3,000 mf/mL, 4 were positive (40%), while 11 of 12 pools (91.7%) scored positive after feeding on a volunteer with 80,000 mf/mL.

### 2.6 Detection of *O. volvulus* infection in wild-caught *Simulium* using microscopy and O-150 LAMP

Of the 22,274 black flies (*S. damnosum*) collected, 9,134 were dissected to compute the entomological indices and the remaining flies were stored in 80% ethanol (Supplementary Table S1). Of the flies stored in ethanol, 12,900 were divided into 129 pools for DNA extraction and LAMP assay. By microscopy, a parity of 24.7% (2,257/9,134), infection rate of 41.2/1,000 parous flies and an infective rate of 11.0/ 1,000 parous flies were observed (Table 4).

A total of 258 sub pools (129 head pools and 129 body pools) were analyzed with the LAMP assay. As per the World Health Organization (42), we assumed a parity of 50% in the fly pools to calculate infection/infective rates per 1,000 parous flies. Out of 129 head pools, 47 (36.4%) were positive, while 114 (88.4%) were positive for either body or head infection or both. The calculated infection and infective rates for the O-150 LAMP were 42.6 (n = 129 pools of 100 flies with 15 negative pools) and 9.0 (n = 129 pools of 100 flies with 82 negative pools) per 1,000 parous flies, respectively (Table 4).

### 2.7 Detection of *L. loa* infection in wild-caught *Chrysops* using microscopy and RF4 LAMP

A total of 7,841 wild-caught *Chrysops* dissected and examined by microscopy (Supplementary Table S2), 257 (3.3%) were infected with *L. loa* (Table 5). The non-MDA site Batouri HD had the highest infection rate of 4.4% (103/2365). Among the HDs receiving MDA, the infection rate ranged from 10/812 (1.2%) in South West 2 to 3.5% (17/485) in North West.

The RF4 LAMP assay using DNA extracted from 1291 wild-caught *Chrysops* flies identified 304 positive flies and an overall infection of 23.5% (Table 5). In the non-MDA site, the infection rate was 26.2% (48/183). Similar levels of infection of 30.2% (88/129) and 31.6% (138/434) were observed in the North West and SW 1 sites. In the Eastern MDA site (Messamena HD) and South West 2 CDTI site, the infection rates were lower; 16.5% (30/183) and 0.5% (1/200), respectively. The RF4 colorimetric LAMP assay was significantly more sensitive than microscopy in detecting *L. loa* infection in wild-caught *Chrysops* (P<0.001) (Table 5, with the exception of the South West 2 CDTI project site where the infection rate detected by the two methods was low (P = 0.105).

### 2.8 Detection of *M. perstans* infection in wild-caught *Culicoides* using Mp419 LAMP

Wild-caught *Culicoides* spp. were identified morphologically and then divided into pools for DNA extraction and pool screening with the Mp419 LAMP assay: *C. milnei*, 181 pools of 10 flies/pool; *C. grahamii*, 225 pools of 50 flies/pool; *C. fulvithorax*, 51 pools of 50 flies/pool; *C. innornatipennis*, 2 pools of 50 flies/pool; and *C. neavei*, 1 pool of 50 flies. Infection was found only in *C. milnei* (0.2%) and *C. grahamii* (0.04%) flies (Table 6).

## 3 Discussion

Sensitive and specific diagnostics are needed for assessing pathogen population changes in both human and vector hosts so that MDA interventions can be certified for achieving elimination goals. However, when prevalence is reduced due to successful control measures, e.g., MDA, monitoring of parasites in human or vector samples by conventional techniques, i.e., microscopy, can be challenging. Recently, a panel of new highly sensitive and rapid LAMP tests with a simple color readout have become available for the specific detection of *O. volvulus* (39), *L. loa* (43) and *M. perstans* (40). The goal of this study was to perform a comprehensive evaluation of the performance of these promising methods by testing samples collected from humans and the filarial nematode vectors.

The O-150 colorimetric LAMP assay was used in comparison to microscopy to identify *O. volvulus* infected individuals from different study sites. In each geographic location, significantly higher prevalences were detected using O-150 LAMP. Yabassi, Loum, and Nkondjock HDs recorded the highest prevalence with LAMP (60.4%, 60.0%, and 31.0%, respectively) while the Melong HD recorded the lowest prevalence of 28.1%. The greater number of positives using LAMP is consistent with the greater sensitivity of LAMP previously reported in populations that have received MDA in which the low sensitivity of microscopy was exacerbated (39, 44). This is also consistent with our observation of far greater number of LAMP positives in the Nkondjock HD, an area of low prevalence resulting from MDA. A study to model the sensitivity of the skin snip method showed that the degree of aggregation of mf in skin increases as worm burden declines (45). As such, it is expected that in settings of hypoendemicity, mf are more aggregated and skin snips becomes less sensitive (46, 47). This explains why skin snipping may fail to diagnose light infections as the chance that a snip contains few mf is increased. Interestingly, we also observed a few samples (10) that were positive for microscopy but negative for LAMP. It is possible that these mf are a different filarial species (e.g., *M. streptocerca*) as demonstrated in other studies (17), and highlights the importance of having a highly specific molecular test for skin snip analyses. The “true” prevalence might be better defined by latent class analysis (41), as it may be closer to the LAMP results; this is being done currently.

To further evaluate the suitability of the LAMP assay to monitor *O. volvulus* infection in vectors, we assayed DNA isolated from experimentally infected *Simulium* black flies. All 16 (100%) pools from the 32 flies engorged on the volunteer were positive by the LAMP. When the assay was further used to analyze pools of wild-caught black flies LAMP detected a similar number of positives compared with dissection and microscopy. It is important to note that the infection and infective rates from pool screening were calculated with an assumed parity of 50% as recommended (42). However, for the >9000 flies dissected we observed just 24.7% parous flies. If we were to use our observed parity to compute the pool screening infection and infective rates, the rates per 1000 parous flies for the LAMP would have generated twice as many positives, and thus LAMP would be more sensitive than microscopy. A higher level of sensitivity of LAMP was also previously observed in studies using pools containing 200 black flies spiked with 0.1 ng *O. volvulus* DNA (48).

The *L. loa* RF4 LAMP assay was evaluated on blood samples collected from 4 different health districts: Yabassi, Loum, Melong and Manjo. Interestingly, both LAMP and microscopy showed a good concordance and a kappa index of 95.6% in all regions. In Yabassi, Loum, and Melong, the prevalence of infection was similar (21.2 – 28.5%), while substantially lower in Manjo (5.9%).

The suitability and sensitivity of LAMP to detect infection in infected *Chrysops* was clearly demonstrated using experimentally infected *Chrysops* which had fed on individuals with either high (>30,000 mf/ml, 94.4% sensitivity) or low parasitemia (<10 mf/ml, 88.9% sensitivity). After ingestion by the vector, *L. loa* microfilariae require 7-14 days to develop into infective stage larvae (49, 50). In experimentally infected *Chrysops* that had fed on a volunteer with a low level of infection, parasites were detected solely in the abdomen as early as day 1 and up to day 7 post infection with the LAMP assay. Parasites were found throughout the flies from day 10 onwards. In contrast, infection was detected in the head, thorax and abdomen on days 1-14 post infection in flies that had fed on an individual with a high parasitemia. The ability of the RF4 LAMP assay to detect all developmental stages of the parasite in flies that had fed on a volunteer with low (10 mf/ml) parasitaemia suggests the suitability of this method for identification of *Chrysops* with extremely low levels of infection that may be missed using microscopy. Significantly higher rates of infection were detected using LAMP, with the East MDA, East non-MDA, South West 1, and North West sites having the highest infection rates with LAMP (16.4%-31.6%) while the South West 2 CDTI project site recorded the lowest infection rate of 0.5%.

Despite more than a decade of onchocerciasis CDTI in the South West 1 CDTI project, infection rates have remained high. This may be due to the persistence of a permanent parasite reservoir. Alternatively, a study by Wanji et al. has identified increased apathy towards ivermectin intake in the study area (51). Low adherence in meso- and hyper endemic areas may exacerbate infection transmission and maybe the result of the fear of side effects to ivermectin (52, 53).

The Mp419 LAMP had excellent sensitivity (96.7%), specificity (94.2%) and Kappa index (89%), when using blood samples, indicating that the LAMP assay is equivalent to microscopy. The utility of the Mp419 LAMP assay was also demonstrated in the *Culicoides* vectors, providing a new tool for entomological screening for *M. perstans*.

In summary, we demonstrate the suitability and usefulness of the panel of simple LAMP tests to detect *O. volvulus*, *L. loa* and *M. perstans* in humans and different vector species under experimental and field conditions. These new tools should facilitate surveillance and control efforts for important filarial diseases.

## 4 Materials and methods

### 4.1 Study sites

**Figure 1:**
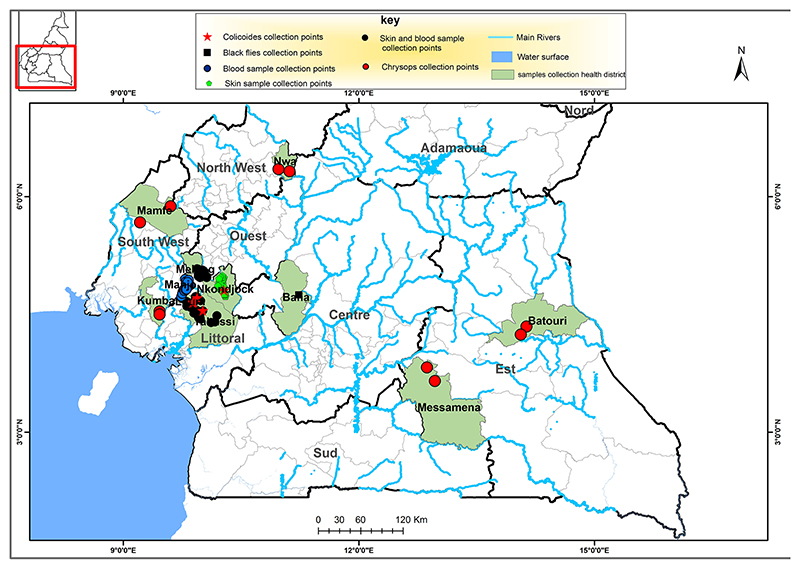
Map of the southern portion Cameroon depicting the regions in which the human and vector samples were collected, as indicated in the key. This map was created using ArcGIS (ArcMap v10.5.1) software by Esri.

The parasitological survey of the study was conducted from the month of May 2020 to January 2021 in the Littoral region of Cameroon, in the Loum (4°42′58″N, 9°44′47″E), Melong (5°7′16″N, 9°57′10″E), Yabassi (4°27′16″N, 9°57′56″E), Nkondjock (4°10′06″N, 10°04′51″E) and Manjo health districts (HDs), belonging to the Littoral 2 Community-Directed Treatment with Ivermectin (CDTI) project (Figure 1). For the skin snip samples, individuals were recruited from; Loum, Melong, Yabassi and Nkondjock HDs. While for blood samples, individuals were recruited from Loum, Melong, Yabassi and Manjo HDs. These HDs have different levels of filarial endemicity and had been under CDTI for over 16 years prior to the study.

The cross-sectional entomological survey was conducted in the Centre region (*Simulium*), South West, North West and East region (*Chrysops*) and the Littoral region (*Culicoides*) in Cameroon. Blackflies were collected in the village of Biatsotsa located on the River Mbam in the Bafia HD, part of the Mbam drainage basin. The Bafia HD belongs to the Centre 1 CDTI project area that, despite over 20 rounds of annual CDTI, is still meso-endemic for onchocerciasis (54).

The South West 1 (kumba HD) and South West 2 (Mamfe HD) are situated in areas of mild *L. loa* endemicity that had received CDTI for more 12-14 years by the time of the study (55). The Eastern and North West project sites are situated in high *L. loa* endemicity areas that had received CDTI for 10 and 9 years, respectively, prior to the study (55, 56).

### 4.2 Study design

The study was comprised of two main parts: parasitological survey and an entomological survey. The parasitological survey involved the use of skin/blood samples to detect skin-dwelling and blood-dwelling mf infection and the entomological survey involved the use of experimentally-infected flies to determine sensitivity and a field phase using wild-caught insects.

### 4.3 Parasitological evaluation

#### 4.3.1 *O. volvulus* (skin dwelling infection)

##### 4.3.1.1 Nodule palpation

The examination for the presence or absence of nodules was done following the WHO standard protocol as previously conducted (51). Consenting participants were examined in a well-illuminated and enclosed room to maintain participant privacy (3); with emphasis being laid on the bony prominences. The number of nodules was recorded and their location marked on anatomical diagrams in the participant recruitment forms.

##### 4.3.1.2 Skin snipping

Skin snipping was done as previously described (51). Prior to skin snipping, the iliac crest of each participant was cleaned with a 70% alcohol pad and allowed to air dry. Two bloodless skin biopsies from the posterior left and right iliac crest of each participant were taken using a sterile 2 mm corneo-scleral punch (CT 016 Everhards 2218-15 C, Meckenheim, Germany). Baneocin antibiotic powder was applied to the snipped areas to prevent infection. The two skin samples from each participant were placed in two separate wells of a 96 well microtiter plate containing 100 uL of saline and incubated at room temperature for a period of 24 hours to allow maximum emergence of mf from the skin. The plates were sealed with parafilm to prevent any spill-over or evaporation of the saline (51). Emerged mf were counted using a light microscope at 10x magnification and expressed per skin snip (51). For each individual, the skin biopsies were placed in the same 1.5 mL Eppendorf tube (Eppendorf AG, Hamburg, Germany) containing 80% ethanol (GAPUMA UK Limited) and stored at -20°C for subsequent DNA extraction and LAMP analysis.

##### 4.3.1.3 Genomic DNA extraction from skin biopsies

DNA from skin biopsies was extracted using the QIAGEN DNeasy Blood and Tissue Kit (Qiagen, Hilden, Germany), following the manufacturer’s instruction. Using sterilized forceps, skin biopsies were placed into 2 mL Eppendorf tubes (Eppendorf AG) containing 160 mL of 1X Phosphate Buffered Saline (Sigma-Aldrich, USA) and 18–20 (1.0–1.3 mm) glass beads (VWR International, Darmstadt, Germany). The skin snips were homogenized at 7000 rpm for 180s using a MagNA Lyser Instrument (Roche Diagnostics GmbH, Mannheim, Germany). After homogenization, the Qiagen protocol was followed and DNA was eluted in 200 μL of elution buffer and stored at -20°C until use for LAMP assays.

#### 4.3.2 *L. loa* and *M. perstans* (blood dwelling infections)

##### 4.3.2.1 Thick Blood Film (TBF) for blood parasites

Diurnal blood collections were performed between 8am-4pm. The thick blood film (TBF) was prepared by spreading 50 mL non-heparinized finger-prick blood on clean, labeled and dry slides covering an area of 1.5×2.5 cm. The smears were allowed to air-dry and then packaged for transport back to the base for staining with 10% Giemsa using standard procedures (25).

##### 4.3.2.2 Parasite identification

The stained smears were read at a 10× magnification using a light microscope by trained technicians. Parasites (*L. loa* and *M. perstans*) were identified using microfilarial identification keys (57). Counts were quantified and expressed as microfilariae per milliliter (mf/mL) of blood.

##### 4.3.2.3 Venous blood collection for molecular detection of blood parasites

A prominent vein was located and disinfected using a cotton swab soaked with 70% alcohol. A sterile 5 mL syringe was used to collect 2 mL of venous blood. The blood was transferred to labelled sterile EDTA tubes. Blood samples were placed in a cooled box containing ice packs and transported to the laboratory for DNA extraction and LAMP assay.

##### 4.3.2.4 Genomic DNA extraction from blood cell pellets

Blood samples were allowed to thaw at room temperature prior to analyses. DNA from blood pellets was extracted using QIAGEN QIAmp DNA Mini Kit (Qiagen, Hilden, Germany), following the manufacturers’ instructions. Blood samples were homogenized with the use of a pipette prior to dispensing 200 μL of the blood samples into the respective labelled 1.5 mL tubes containing 20 μL of Protease. To these tubes, 200 μL of Buffer AL was added and thoroughly homogenized by pulse vortexing for 15 seconds and incubated at 56°C for 10min. Following incubation, 200 μL of 96% ethanol was added and vortexed thoroughly before pipetting into a QIAmp DNA Mini spin column which was placed in a 2 mL collection tube. The remaining steps were performed as described above for skin snip DNA extraction.

### 4.4 Entomological evaluation

#### 4.4.1 Collection and laboratory maintenance of experimentally fed (*Simulium*, *Chrysops* and *Culicoides*) flies

##### 4.4.1.1 Collection and laboratory maintenance of experimentally fed *Simulium damnosum* flies

Female blood-seeking *S. damnosum* flies allowed to take blood on a consented microfilaridermic volunteer (50 mf/mL) were captured using *Simulium* rearing tubes. and transported to the laboratory (58). Upon arrival in the laboratory, *Simulium* flies were maintained in captivity under controlled experimental conditions in the insectarium for a period of 10 days to allow ingested microfilariae to mature into infective stage larvae (L3). During this period, flies were fed with sterile 15% sucrose solution and maintained at a temperature of 23–28°C and 79–80% relative humidity (58). Four flies were frozen at -20°C on day 3, 4, 5, 6, 7, 8, 9 and 10 post infection. At the end of the experiment, the flies were distributed into pools of 2 flies/pool for each day post infection and DNA was extracted from each pool and subjected to the O-150 LAMP assay for detection of *O. volvulus* infection.

##### 4.4.1.2 Collection and laboratory maintenance of experimentally fed *Chrysops* flies

After providing informed consent, microfilaremic volunteers allowed *Chrysops* flies to take blood which were caught using 50 mL Falcon tubes (Corning, USA) (59). To produce experimental infections in flies, two batches of 18 flies were each fed on either a microfilaremic volunteer with low (<10 mf/mL) microfilaremia (Lot 1) or with high (>30,000 mf/mL of blood) microfilaremia (Lot 2). Flies were maintained in the insectarium (23–28°C; relative humidity between 79–80%(60)) for up to 14 days to monitor larval development to L3 stage. Flies were fed daily with sterile 15% sucrose solution. From each lot, two flies were frozen at -20°C on day 0 (<7 hours post infection), 1, 4, 6, 7, 10, 11, 12 and 14 post infection. At the end of the experiment, the flies were dissected into head, thorax and abdomen sections; DNA was extracted from each body part for analysis by RF4 LAMP assay.

##### 4.4.1.3 Collection and laboratory maintenance of experimentally fed *Culicoides* flies

Collections were done each working day from 6pm-6am using rectangular cage traps (2x2x2m). *M. perstans* microfilaremic volunteers were seated with their legs exposed to the knee level under a rectangular netting cage trap. The cage was raised for 10-15 minutes to allow contact between host and midges and then lowered to trap the attracted midges. After 15 minutes, the *Culicoides* spp. within the cage were aspirated and blown into labeled 50 mL Falcon tubes filled 3/4 with plaster of Paris (POP) which served as an absorbent layer at the bottom of the tubes to retain moisture. Once transported to the insectarium [23-28°C; relative humidity 79-80% (60)], they were monitored daily for 12 days (time for the mf to develop to L3 stage). The flies were maintained singly in 50 mL Falcon tubes and fed daily with sterile 15% sucrose solution soaked in cotton wool. At intervals of 24 hours, a drop or two of distilled water was added using a 10 mL syringe to the POP to replenish moisture content in the tube. Flies were pooled (max. 10 flies) into 0-2, 3-5, 6-8 and 9-12 days post infection groups. The flies were identified, and DNA was extracted from each pool for detection with the Mp419 LAMP assay.

#### 4.4.2 Field collection and dissection of wild vectors (*Simulium*, *Chrysops* and *Culicoides*) flies

##### 4.4.2.1 Field collection of wild Simulium flies

Black flies were collected in June 2016 by the human landing collection method. The catch was done daily on an hourly basis between 7:00 to 18:00 for a period of 4 days. Female *Simulium* flies coming for a blood meal were caught just as they landed using an aspirator as described in other studies (58).

###### 4.4.2.1.1 Dissection of female Simulium flies

Captured female blackflies were anaesthetized with chloroform and identified for species and counted. Then the flies were dissected in physiological saline under a dissecting microscope by entomology experts who distinguished parous (very reduced or clear malpighian tubules and less or no fat bodies) from nulliparous (large or opec malpighian tubules and more fat bodies) flies as described (61). The abdomen, thorax and head of parous flies were further dissected and examined for the presence of *Onchocerca*. Larvae were identified by species, counted and recorded by larval stage (L1, L2, L3). Undissected flies were pooled in batches of 100 into 80% ethanol for DNA extraction and LAMP pool screening.

###### 4.4.2.1.2 Purification of DNA from Simulium flies

Each pool of whole flies, fly heads or bodies was weighed (average weight for head and body pools was 17 mg and 100 mg, respectively) and placed in a 1.5 mL Eppendorf tube. Total DNA was extracted using the Zymo Research Genomic DNA Tissue MiniPrep Kit (Epigenetics Company, USA) following manufacturer’s protocol; for pools of bodies (tissue mass >25 mg), the volumes of digestion buffer and proteinase K were doubled. All samples were eluted with 200 μL and the DNA stored at -20°C until use.

##### 4.4.2.2 Field collection of wild *Chrysops* flies

Insect collections were performed between 7:00 and 18:00 from the August to October 2014 for a period of 5 days/ community (62). Trained collectors dressed in protective clothing to prevent insect bites and stationed near a wood fire caught blood-seeking female flies using sweep nets; fly numbers per hour were recorded. At the end of each session, wild-caught flies were randomly separated into three groups: 1) control group dissected to check for parity, retaining 138 nulliparous flies for the RF4 LAMP assay; 2) group for dissection and microscopy; 3) stored in 80% alcohol for DNA extraction and LAMP analysis.

###### 4.4.2.2.1 Dissection of wild Chrysops flies

In the field laboratory, wild *Chrysops* were dissected in 0.9% NaCl under a dissecting microscope. The head, thorax and abdomen of each fly were separated and the abdomens were teased gently to pull out the ovarioles to determine parous status of the flies. Parous flies were further dissected to find *L. loa* larvae, counted and the infection rates calculated as described (63, 64).

###### 4.4.2.2.2 Purification of DNA from Chrysops flies

Individual *Chrysops* flies were crushed using micro pestlein Eppendorf tubes containing 95 μL of water, 95 μL 2 X digestion buffer (Zymo Research Genomic DNA Tissue^™^ MiniPrep Kit) and 10 μL Proteinase K, incubated at 55°C for 1-3 hours followed by DNA extraction according to manufacturer’s protocol. DNA was eluted with 200 μL of elution buffer and stored at -20°C until use.

##### 4.4.2.3 Field collection of Culicoides flies

Collections of midges were carried out using Centers for Disease Control miniature black UV-light traps (Model 512, John W. Hock Company, Gainesville, USA). UV-light traps set at strategic positions around human dwellings at each of the seven sites. Midges were collected hourly between 6:00 and 18:00. The contents of each trap were emptied hourly using aspirators into labelled plastic cups containing 80% alcohol and then transported to the laboratory for *Culicoides* identification.

###### 4.4.2.3.1 Morphological identification of adult *Culicoides* species

Morphological identification of *Culicoides* species was done based on keys through the examination of the wing pigmentation pattern under a dissecting microscope (65, 66). After identification, the flies were pooled (10 or 50 flies/pool) according to species for DNA extraction.

###### 4.4.2.3.2 Purification of DNA from Culicoides flies

DNA from Culicoides flies was extracted using the QIAGEN QIAmp DNA Mini Kit (Qiagen, Hilden, Germany), following the manufacturer’s instruction. Flies were placed into 2 mL Eppendorf tubes containing 80 μL of 1X Phosphate Buffered Saline (SIGMA-ALDRICH,USA) and 18–20 (1.0–1.3 mm), glass beads (VWR International, Darmstadt, Germany) and homogenized at 4000 rpm for 90 seconds using a MagNA Lyser Instrument (Roche Diagnostics GmbH). After homogenization, 180 μL of ATL buffer and 20 μL of proteinase K were added into the homogenate and incubated at 56°C overnight. Following the overnight incubation, samples were processed per the protocol and the DNA was eluted with 200 μL of elution buffer. The eluted DNA was stored at -20°C until use for LAMP assays.

### 4.5 LAMP assay to detect *O. volvulus*, *L. loa* and *M. perstans*

To prevent cross-contamination, all sufaces were cleaned with 10% bleach solution and filter tips were used. All tubes are tightly closed and never opened after amplification to avoid contaminating the work area. The *O. volvulus* tandem repeat region O-150 was targeted in the study, the colorimetric O-150 LAMP assay was performed with some modifications (39). The *L. loa* LAMP targeting the RF4 family repeat was performed as previously published (43). The *M. perstans* LAMP targeting the Mp419 repeat was performed as published (40), with some modifications. Primer sequences and reaction components are detailed in Tables S1 and S2, respectively. Reactions were performed in a GeneAmp^®^, PCR System 9700 Thermal Cycler (Applied Biosystems, Foster city, USA). Samples were considered positive for the indicated filarial nematode if a color change from pink to yellow was observed (Figure S1), while non-template controls (molecular biology grade H_2_O) remained pink.

### 4.6 Data processing and analysis

Data collected and compiled on paper record sheets were entered into Microsoft Excel 2010 and then exported to GraphPad Prism version 9.0 for subsequent analyses. Contingency tables were used to express the relationship between variables. Fischer’s Exact test was used to compare proportions with P ≤ 0.05 considered significant.

LAMP assay performance characteristic values (sensitivity, specificity, positive predictive value (PPV), negative predictive value (NPV) and Kappa index) standard methods employing 2x2 contingency tables for evaluation of diagnostic tests were used. Microscopy was used as the “gold standard”. The 95% confidence intervals for all values were calculated as previously described (67).

Infection and infective rates for *Simulium* flies using microscopy were computed as previously described (68). The infection and infective rates from pool screening of *Simulium* and *Culicoides* flies were computed using the algorithm described by Katholi and colleagues (69).

$$P=1-\sqrt[{n}]{\frac{k}{m}}$$

Where **m** = number of pools, **n** = size of pool, **k** = number of negative pools and *P* = prevalence of infection.

The infection rate for *Chrysops* was determined as the proportion of infected flies to the total number of flies dissected as previously described (59).

## Supplementary Material

Supplementary Material

Supplementary Table S1

Supplementary Table S2

## Figures and Tables

**Table 1:** Prevalence of *O. volvulus*, *L. loa*, and *M. perstans* infections in the health districts as determined by microscopy and LAMP assays.

LAMP Assay	Health District	Total Screened	Microscopy No. Positive (%)	LAMP No. Positive (%)	P- value*	Kappa Statistic (95% CI)
**O-150 (*O. volvulus*)**	**Loum**	70	23 (32.9)	42 (60)	**0.002**	0.49 (0.324 -0.660)
	**Yabassi**	164	62 (37.8)	100 (61.0)	**<0.0001**	0.47 (0.350 -0.585)
	**Melong**	210	33 (15.7)	59 (28.1)	**0.003**	0.54 (0.406 - 0.668)
	**Nkondjock**	226	20 (8.8)	70 (31.0)	**<0.0001**	0.30 (0.183- 0.425)
**RF4 (*L. loa*)**	**Loum**	22	5(22.7)	5(22.7)	1.000	1.00(1.00-1.00)
	**Yabassi**	165	44(26.7)	47(28.5)	0.805	0.92 (0.86- 1.00)
	**Melong**	99	22(22.2)	21(21.2)	1.000	0.97 (0.91- 1.00)
	**Manjo**	186	11(5.9)	11(5.9)	1.000	1.00(1.00-1.00)
**Mp419 (*M. perstans*)**	**Loum**	68	48(70.6)	49(72.1)	1.000	0.96 (0.89-1.00)
	**Yabassi**	328	169(51.5)	187(56.8)	0.183	0.81 (0.75-0.87)
	**Melong**	137	26(19)	25(18.2)	1.000	0.93(0.85-1.00)
	**Manjo**	192	2(1.0)	4(2.1)	0.685	0.66 (0.23-1.00)

* Fisher’s Exact Test.

Bold text indicates P-value ≤ 0.05.

**Table 2:** Performance characteristics of the three LAMP methods evaluated using microscopy as reference method.

	O-150 LAMP	RF4 LAMP	Mp419 LAMP
**Sensitivity % (95% CI)**	92.8 (87.17-96.02)	97.6 (91.54-99.57)	96.7 (93.69-98.34)
**Specificity % (95% CI)**	73.1 (69.20-76.71)	99.0 (97.39-99.6)	94.2 (91.70-95.93)
**PPV % (95% CI)**	47.2 (41.37-53.17)	95.2 (88.39-98.13)	89.5 (85.15-92.59)
**NPV % (95% CI)**	97.5 (95.45-98.63)	99.5 (98.14-99.91)	98.3 (96.61-99.12)
**Kappa % (95% CI)**	48.5 (42.2-54.9)	95.6 (92.1-99.1)	89.1 (85.7-92.6)

**Table 3:** *L. loa* infection rates in experimentally infected *Chrysops* determined by RF4 LAMP.

Level ofParasitemia*	Infection Status		Total
	Positive (%)	Negative (%)	
Low	16 (88.9)	2 (11.1)	18
High	17 (94.4)	1 (5.6)	18
Total	33 (91.7)	3 (8.3)	36

* Parasite load in volunteers who allowed vectors to take a blood meal.

**Table 4:** *O. volvulus* infection rates in wild-caught *Simulium* flies as determined by microscopy and O-150 LAMP.

Parameter	Total screened (pools)*	MICROSCOPY	O-150 LAMP	P- value
Infection rate (%)	129	4.12	4.26	0.929
Infection rate per 1000 parous flies	129	41.2	42.6	
Infective rate (%)	129	1.1	0.9	0.483
Infective rate per 1000 parous flies	129	11.0	9.0	

* 100 fl ies (heads or bodies) per pool.

**Table 5:** Natural infection rates of wild-caught *Chrysops* in various study sites determined by microscopy and LAMP methods.

Study Sites (Years of MDA)	Microscopy		Colorimetric LAMP		P-value^#^
	Total Screened	No. Positive (%)	Total Screened	No. Positive (%)	
East MDA * (9)	2365	61 (3.3)	183	30 (16.4)	<0.0001
East non-MDA (0)	1861	103 (4.4)	183	48 (26.2)	<0.0001
South West 1 * (15)	2318	66 (2.8)	434	137 (31.6)	<0.0001
South West 2 (13)	812	10 (1.2)	200	1 (0.5)	0.105
North West * (10)	485	17 (3.5)	291	88 (30.2)	<0.0001
Total	7841	257 (3.3)	1291	304 (23.5)	<0.0001

* East MDA: Messamena Health District.

East non-MDA: Batouri Health District

North West: Nwa Health District

South West 1: Kumba Health District

South West 2: Mamfe Health District.

# Fischer’s Exact test.

**Table 6:** Natural infection rates in different species of wild-caught *Culicoides* spp. determined by Mp419 LAMP.

Species	No. of Flies	No. of Pools	No. Positive	Infection Rate (%)
*C. grahamii*	11250	225*	2	0.04
*C. milnei*	1810	181^#^	2	0.2
*C. fulvithorax*	2550	51*	0	0
*C. innornatipennis*	100	2*	0	0
*C. neavei*	50	1*	0	0
**Total**	15670	460	4	

* 50 flies per pool

# 10 flies per pool.

## Data Availability

The original contributions presented in the study are included in the article/Supplementary Material, further inquiries can be directed to the corresponding authors.

## Abbreviations

BIP: Backward inner primer
Bst: *Bacillus sterothermophillus*
CDTI: Community Directed Treatment with ivermectin
FIP: Forward inner primer
HD: health district(s)
LAMP: Loop mediated isothermal ampli fication
L1: Larval stage one
L2: Larval stage two
L3: Larval stage three (infective larvae)
MDA: Mass Drug Administration
Mf/mf: Microfilaria(e)
PIR: Potential Infective Rate
RF4: Repeat Family 4
