# Supplementary material for: Application of loop mediated isothermal amplification (LAMP) assays for the detection of Onchocerca volvulus, Loa loa and Mansonella perstans in humans and vectors
Source: Front Trop Dis. Author manuscript; Available in PMC 2023 Jan 21. (PMC7614089; doi:10.3389/fitd.2022.1016176)
Supplement: Supplementary Material [file EMS160015-supplement-Supplementary_Material.PDF]

Supplementary Material

Supplementary Methods

Table S1. Primer sequences (5'-3') for the O-150, RF4 and Mp419 LAMP assays

Primer	O-150	RF4	Mp419
**FIP**	TGATGACCTATGACCCTAATCTCAA-CGAATATTTTTCTTAGGACCCAAT	CGACGTCTTCACAAGGTAAGCC-GTTTAGCCTTGAGTTAGGATC	TGTGAGCACATTTCAGTAAGT-GATGAAATCCACTAAATTCWC
**BIP**	TGAAAATGCGTTTTTCGCCGGG-GTCCTAAGAAAAATATTCGACTA	AGGACACAGAGTAAAATTTACCGCT-CGATTTYCTACTCGTTATTCTTCAA	GGATTCTTTCTAAAAGTTGAG-GATCGATTTCGTTAAAAACAGY
**F3**	TGGAAATTCACCAAAATATGGT	TCTTTCYTTTTATCGAGTCGTT	ACAGTTGATTATTTGAAGGTGCTR
**B3**	GGGTACGTACCTTCAAACTG	AACAGCYTTTGACTCACG	AYAATGATTATTTYTAAAGAATC
**LF**	AACGGGTACATACATTC	TTAATTAAAGTTCTGCT	AGACTTGATTACTGTTTGG
**LB**	AATCGCCGTGTAAATGTGG	TACAGAGTTGATCAGTAGG	ACAATTTGGTAATCGCTTAAACTG

Table S2. Master mixes per sample for the O-150, R4 and Mp419 LAMP assays

Component	O-150	RF4	Mp419
2X Warm Start Colorimetric Master Mix (New England Biolabs, Inc., USA)	10 µL	12.5 µL	10 µL
100 µM FIP	1.6 µM	1.6 µM	1.6 µM
100 µM BIP	1.6 µM	1.6 µM	1.6 µM
100 µM F3	0.2 µM	0.2 µM	0.2 µM
100 µM B3	0.2 µM	0.2 µM	0.2 µM
100 µM LF	0.4 µM	0.4 µM	0.4 µM
100 µM LB	0.4 µM	0.4 µM	0.4 µM
400 mM Guanidine Hydrochloride	2 µL	--	2 µL
DNA/No template control	2 µL	2 µL	2 µL
Incubation temperature (minutes)	66 °C (60)	61 °C (40)	63 °C (40)
Total volume	20 µL	25 µL	20 µL

Supplementary Figures

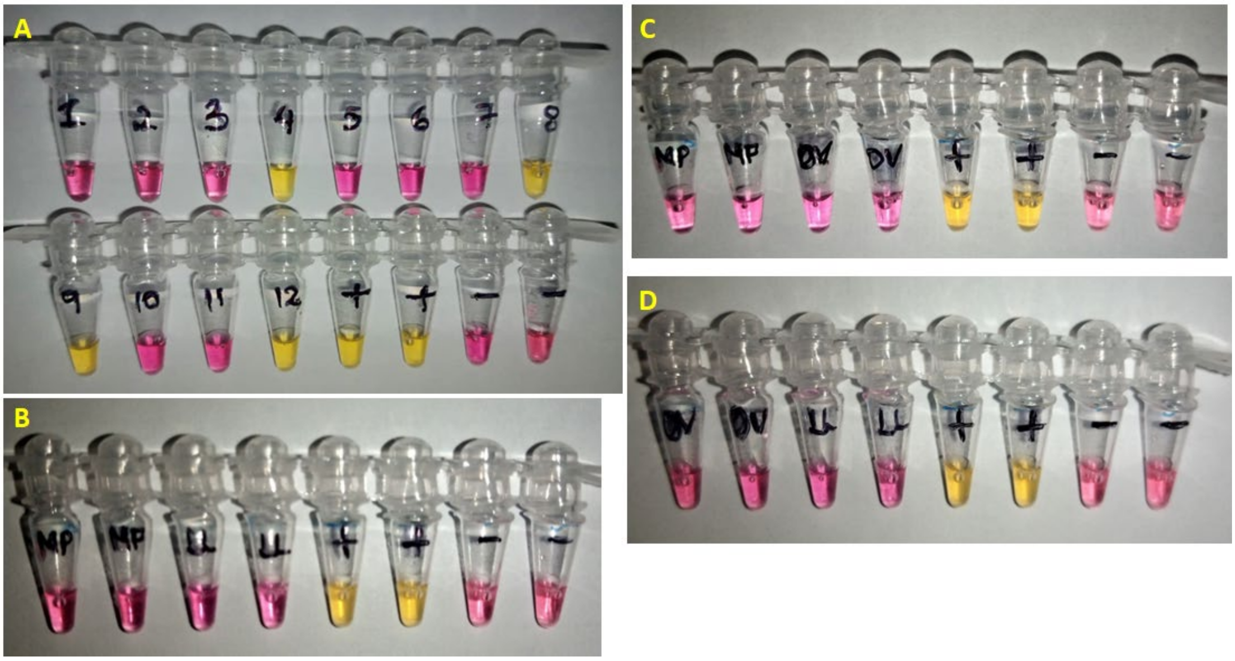

Figure S1. Examples of species specificity of the three filarial LAMP assays. A) *Onchocerca volvulus* LAMP assay using DNA extracted from skin snips of infected and uninfected volunteers. B) O-150 LAMP using DNA from *M. perstans* (MP) and *L. loa* (LL) infected volunteers. C) RF4 LAMP assay using DNA from *M. perstans* and *O. volvulus* (OV) infected volunteers. D) Mp419 LAMP assay using DNA from *O. volvulus* and *L. loa* infected. (+) = Positive control (yellow). (-) = Non template controls of one tube containing molecular biology grade water and another containing elution buffer (pink), Amplification was carried out using the colorimetric master mix (New England Biolabs).
